# Estimating the disability adjusted life years associated with COVID-19 in Iran for the first 2 years of the pandemic

**DOI:** 10.3389/fpubh.2023.1303549

**Published:** 2024-01-11

**Authors:** Saied -Bokaie, Salman Daneshi, Alireza Bahonar, AliAkbar Haghdoost, Eshagh Barfar, Declan Patrick Moran

**Affiliations:** ^1^Department of Food Hygiene and Quality Control, Division of Epidemiology and Zoonoses, Faculty of Veterinary Medicine, University of Tehran, Tehran, Iran; ^2^Department of Public Health, School of Health, Jiroft University of Medical Sciences, Jiroft, Iran; ^3^Research Center for Modeling in Health, Institute for Future Studies in Health, Kerman University of Medical Sciences, Kerman, Iran; ^4^Health Promotion Research Center, Zahedan University of Medical Sciences, Zahedan, Iran; ^5^School of Public Health, University College Cork, Cork, Ireland

**Keywords:** COVID-19, pandemic, burden of disease, Iran, DALYs

## Abstract

**Background:**

The World Health Organization (WHO) declared a pandemic of coronavirus disease 2019 (COVID-19), caused by severe acute respiratory syndrome coronavirus 2 (SARS-CoV-2), on 11 March 2020. Disability-adjusted life years (DALYs) combine the impact of morbidity and mortality, allowing for comprehensive comparisons of the population. The purpose of this study was to estimate DALYs due to COVID-19 in Iran for the first 2 years of the pandemic.

**Methods:**

DALYs were estimated as the sum of Years of Life Lost (YLLs) and Years Lived with Disability (YLDs) associated with COVID-19 in Iran from 19 February 2020 to 20 March 2022. The life expectancy for COVID-19 YLL estimations was based on the Global Burden of Disease (GBD) 2019 study.

**Results:**

There were 15,639,243 outpatients and 1,170,602 hospitalized confirmed cases, of which 120,965 deaths were as a direct result of COVID-19. DALYs were estimated to be 2,376,552. Overall, YLL contributed to 99.34% of the DALYs, while the remaining 0.66% was attributed to YLD.

**Conclusion:**

COVID-19 had a significant impact on population health in Iran during the first 2 years of the pandemic; this study provides a comprehensive depiction of COVID-19’s burden and is helpful for comparing its impact with other diseases in the population and across populations.

## Background

A novel coronavirus (COVID-19) emerged in Wuhan (Hubei, China), in December 2019 and spread to the most regions throughout the world (1) with a variety of clinical signs (2) and caused countries to face large numbers of infected people and created challenges in social, economic, and health areas. On 11 March 2020, the World Health Organization (WHO) declared COVID-19 a pandemic. In Iran, on 19 February 2020 (3), the first infection of COVID-19 was reported in Qom, it was then observed in Tehran and Gilan, and in less than 2 weeks, cases were recorded in all provinces of the country (4). Key strategies to combat the pandemic, which were implemented in many countries including Iran, were national mobilization efforts, which included smart physical distancing, development of laboratories, early diagnosis and rapid identification of sources of infection, contact tracing, wearing face masks, washing hands, and active follow-up of the patient’s relatives (5). Countries acted in different ways to fight COVID-19, so the burden of disease was varieties in countries (6). The interventions implemented in Iran were a combination of changing people’s behavior, improving the level of personal hygiene, creating physical distance, screening, active disease detection and isolation of positive cases (4). The health impact of COVID-19 was different according to age and sex (5) and also has differed across countries; however, estimation is important in assisting health policy decision-making. Estimating the direct health impact of COVID-19 and facilitating comparisons with other diseases and injuries to understand and quantify the combined impact of mortality and morbidity of disease is a key step when standardizing comparisons across countries (7).

In this regard, Burden of Disease frameworks facilitate estimating the health impact of diseases to be translated into a single measure, such as Disability-Adjusted Life Years (DALYs), which is one of the most frequently used health measures to determine the effectiveness of social and systematic interventions. The DALYs concept was developed by World Bank for estimation of the burden of disease resulting from diseases, injuries, and risk factors for comparing health across countries (8). Since the DALYs is a metric to measure the impact of health problems that cover diseases, death, and injuries and shows health problems from a macro perspective for societies and is frequently used (9). The purpose of this study was to estimate the burden of COVID-19 in Iran for the first 2 years of the pandemic.

## Methods

### Target population and setting

This was a descriptive-analytical study. The target population of this study was the total population of Iran in the period from 19 February 2020 to 20 March 2022 according to age and sex groups (5).

COVID-19 affected health through two main pathways: directly, as a new infectious disease; and indirectly, as a risk factor for other diseases, for example, through increases in mental health issues due to national lockdowns or delays to diagnosis and follow-ups treatment of other diseases through restrictions which were necessary to support healthcare service delivery throughout the pandemic period. For this study, we calculated direct burden estimates in three steps: Firstly, the impact of morbidity was estimated in terms of years lived with disability (YLD), secondly years of life lost due to premature mortality (YLL) were estimated and finally, DALYs were quantified by summing YLD and YLL.

### Data sources

Data in this study includes deaths, hospitalized and outpatient COVID-19 cases confirmed through RT-PCR tests. Data on death and hospitalization were received from the Center for Communicable Disease Control (CDC) of Iran’s Ministry of Health and Medical Education (MoHME) that were identified as those where the underlying, or any contributory, cause of death was coded using the emergency ICD-10 codes U07.1 based on guidance from the WHO (10). Outpatient data were estimated according to samples from a number of cities (including Kerman, Mashhad, Chabahar, Rafsanjan, Ghom, Rudbar, Jahrom, Jiroft, Kahnuj, Ghale ganj, Manujan) in the country. All data including hospitalization (in traditional hospital care) and outpatient confirmed cases, recovered cases, and deaths due to COVID-19 are available publicly.

### Years of life lost

Years of life lost (YLL) is calculated as the summation of the number of deaths multiplied by the average remaining life expectancy at the time of death. GBD 2019 reference life tables were used to estimate COVID-19 YLL for each sex separately (7). Mortality data included underlying/initial cause of death due to COVID-19 which was independently stratified by sex and age groups for the period of the study.

Equation 1 was used for calculation:


(1) $$YLL=\frac{NCe^{\left(ra\right)}}{{\left(\beta +r\right)}^{2}}\left[\begin{array}{l} e^{-\left(\beta +r\right)\left(L+a\right)}\left[-\left(\beta +r\right)\left(L+a\right)-1\right]\\ -e^{-\left(\beta +r\right)a}\left[-\left(\beta +r\right)a-1\right] \end{array}\right]$$

In this formula:

N = number of deaths due to COVID-19 or with COVID-19 by age and sex.

L = standard life expectancy of the deceased at the same age and sex.

R = the discount rate, which is equal to 0.03.

Β = beta rate in calculating age value, which is equal to 0.04.

C = constant number is 0.1658.

### Years lived with disability

Years lived with disability (YLD) is calculated as the summation of the number of COVID-19 cases multiplied by the average duration until recovery or death and the disability weight. Based on the study of Mirzaee et al. (11), the duration of COVID-19 (6) was 7.79 and 14 days for outpatients and hospitalized patients, respectively; which were based on 85% of patients experiencing signs and symptoms for 14 days. The disability weight is a necessary variable for YLD estimation, categorizations were considered as follows; outpatient (mild and moderate) and hospitalized (severe) patients, 0.051, 0.133, respectively (6). Equation 2 was used to calculate it:


(2) $$YLD=I\times DW\{\frac{NCe^{\left(ra\right)}}{{\left(\beta +r\right)}^{2}}\left[\begin{array}{l} e^{-\left(\beta +r\right)\left(L+a\right)}\left[-\left(\beta +r\right)\left(L+a\right)-1\right]\\ -e^{-\left(\beta +r\right)a}\left[-\left(\beta +r\right)a-1\right] \end{array}\right]\}$$

In this formula:

I = the number of new cases of COVID-19.

L = duration of that disease or complication.

DW = the weight of that disability or complication.

R = the discount rate, which is equal to 0.03.

Β = the contractual rate in calculating the age value, which is equal to 0.04.

C = a constant number of 0.1658.

e = Napier’s constant is equal to 2.718.

### Disability adjusted life years

Disability adjusted life years (DALYs) are calculated as the summation of YLLs and YLDs, one DALY represents the loss of the equivalent of 1 year of healthy life. The following formula is used to calculate: DALY = YLL + YLD (13).

### Statistical analysis

For ease of comparison with previous studies, the YLLs, YLDs and DALYs were also estimated per 100,000 population.

### Sensitivity analysis

Sensitivity analysis was employed to estimate the impact of alterations in certain parameters of the model on the study results. The uncertainty intervals (UIs) from the GBD 2019 life tables were used to calculate and formulate the upper and lower intervals of our YLL (12). For UI relating to YLD, we used the respective disability weight UIs from the GBD 2019 study for infectious diseases of the lower respiratory tract and the European Disability Weight study. Additionally, durations were informed by a previous study with the minimum and maximum values being 0 and 0.14 for mild to severe, respectively (11). These UIs were subsequently used to estimate the upper and lower estimates of the DALYs.

### Ethical considerations

Ethical approval for this study was obtained from Jiroft University of Medical Sciences: (code: IR.JMU.REC.1400.036).

## Results

### Mortality and morbidity due to COVID-19

During the study period, 15,639,243 outpatients and 1,170,602 hospitalized cases were observed based on confirmed diagnostic criteria, of which 120,965 people died.

### Years of life lost

**Figure 1:**
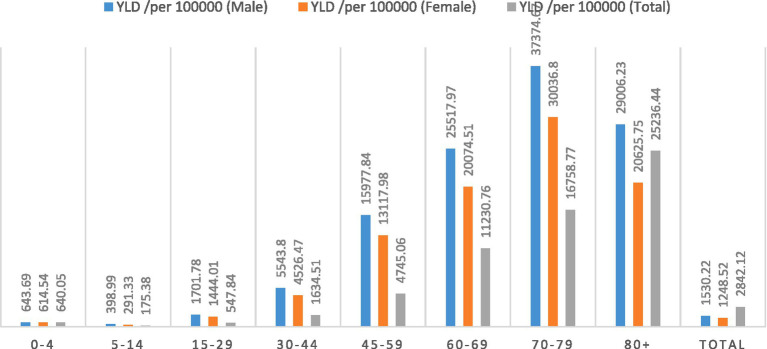
Years of life lost (YLLs) per 100,000 population due to COVID-19 based on age and sex groups in Iran during 2020–2022.

The total COVID-19 YLL was 2,361,066, UI 95% (2,381,165–2,339,160) equivalent to 2842.12 YLL per 100,000 population. In the male population, there were 1,300,211 (95% UI 1288140.45–1311285.75) YLL, equivalent to 1530.22 YLL per 100,000. In the female population, there were 1060855.68 (95% UI 1051019.58–1069088.03) YLL, equivalent to 1248.52 YLL per 100,000 (the number of YLLs is shown in Table 1). In both sex groups, the highest YLL number was seen in the age group 45–59 years and the lowest YLL was seen in the age group 5–14 years. Figure 1 shows the results according to different age and sex groups separately in per 100,000 population.

**Table 1:** Years of life lost (YLLs) due to COVID-19 based on age and sex groups in Iran during 2020–2022.

	Male		Female		Total	
Age groups	YLLs	YLLs/per 100,000	YLLs	YLLs/per 100,000	YLLs	YLLs/per 100,000
0–4	24575.43	643.69	22404.73	614.54	46980.16	640.05
5–14	13518.19	398.99	9388.605	291.33	22906.801	175.38
15–29	54818.95	1701.78	45272.632	1444.01	100091.582	547.84
30–44	209776.18	5543.8	166210.48	4526.47	375986.66	1634.51
45–59	344807.45	15977.84	280165.19	13117.98	624972.64	4745.06
60–69	306510.6	25517.97	256487.4	20074.51	562,998	11230.76
70–79	191924.6	37374.67	172746.2	30036.8	364670.8	16758.77
80+	154279.5	29006.23	108179.5	20625.75	262,459	25236.44
Total	1,300,211	1530.22	1060855.68	1248.52	2,361,066	2842.12

### Years lived with disability

**Figure 2:**
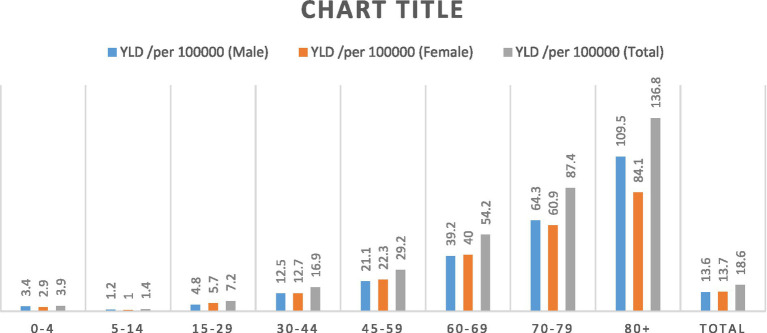
Years lived with disability (YLDs) per 100,000 population due to COVID-19 based on age and sex groups in Iran during 2020–2022.

The number of COVID-19 YLD was 15485.9 (95% UI 10139.26–18656.20), equivalent to 18.6 YLD per 100,000 population. It was 7870.1 (95% UI 5144.32–9551.41) equivalent to 13.6 YLD per 100,000 in the male population and 7,615.8 (95% UI 4994.94–9104.80) equivalent to 13.7 YLD per 100,000 in the female population. The highest YLD in both sex groups was in the age group 30–44 years followed by the 45–59 age group. The lowest YLD was in the age group 0–15 years (number of YLD showed in Table 2). Figure 2 shows the results according to different age and sex groups separately per 100,000 population.

**Table 2:** Years lived with disability (YLDs) due to COVID-19 based on age and sex groups in Iran during 2020–2022.

	Male		Female		Total	
Age groups	YLDs	YLDs/per 100,000	YLDs	YLDs/per 100,000	YLDs	YLDs/per 100,000
0–4	159.5	3.4	127.2	2.9	286.7	3.9
5–14	104.9	1.2	83.3	1.0	188.2	1.4
15–29	631.9	4.8	688.4	5.7	1320.3	7.2
30–44	1995.8	12.5	1912.9	12.7	3908.7	16.9
45–59	1919.4	21.1	1936.5	22.3	3855.9	29.2
60–69	1318.4	39.2	1399.3	40.0	2717.7	54.2
70–79	926.4	64.3	975.7	60.9	1902.1	87.4
80+	813.8	109.5	609.5	84.1	1423.3	136.8
Total	7870.1	13.6	7615.8	13.7	15485.9	18.6

### Disability adjusted life years

**Figure 3:**
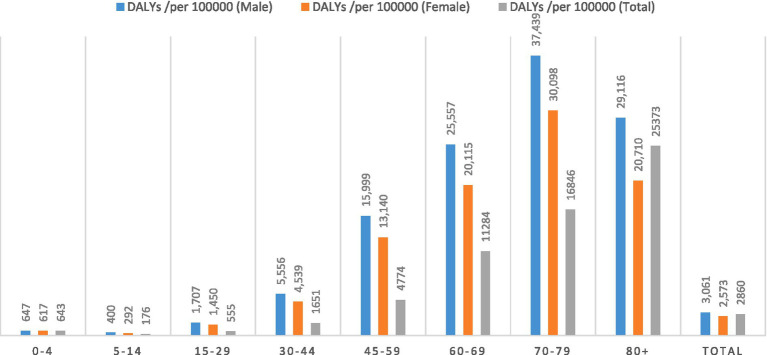
Disability adjusted life years (DALYs) per 100,000 population due to COVID-19 based on age and sex groups in Iran during 2020–2022.

We estimated DALYs due to COVID-19 of 2,376,552 (95% UI 2341876.0–2390969.4), equivalent to 2,860 per 100,000 population; 1,308,081 (95% UI 1289497.5–1316188.6), equivalent to 3,061 years lost per 100,000 males and 106,847 (95% UI 1052378.6–1074780.8) equivalent to 2,573 years lost per 100,000 females. The highest DALYs in both sexes was in the age groups of 45–59 and 60–69, and also in both sexes, the lowest amount was in the age group of 5–14 and 0–4 years (number of DALY showed in Table 3). In Figure 3 the results are given separately according to different age and sex groups in per 100,000 population.

**Table 3:** Disability adjusted life years (DALYs) due to COVID-19 based on age and sex groups in Iran during 2020–2022.

	Male		Female		Total	
Age groups	DALYs	DALYs/per 100,000	DALYs	DALYs/per 100,000	DALYs	DALYs/per 100,000
0–4	24,735	647	22,532	617	47,267	643
5–14	13,623	400	9,472	292	23,095	176
15–29	55,451	1,707	45,961	1,450	101,412	555
30–44	211,772	5,556	168,123	4,539	379,895	1,651
45–59	346,727	15,999	282,102	13,140	628,829	4,774
60–69	307,829	25,557	257,887	20,115	565,716	11,284
70–79	192,851	37,439	173,722	30,098	366,573	16,846
80+	155,093	29,116	108,789	20,710	263,882	25,373
Total	1,308,081	3,061	1,068,471	2,573	2,376,552	2,860

## Discussion

The study of the burden of diseases provides data to assist decision-making in relation to determining priorities, measuring the effectiveness of investments, quantifying various dimensions of social development, determining intervention strategies in the present and future, and the field of prevention, treatment, and rehabilitation for researchers, policymakers, and community managers (6). DALYs have been estimated in many countries using data over varying time durations. Although a number of studies have documented the burden of COVID-19 disease at local scales in Iran, there is no study at the national level. Therefore, this study was designed to estimate the burden of COVID-19 in Iran using DALYs at the national level.

The overall DALYs were 2,376,552 (95% UI 2341876.0–2390969.4) equal to 2842.12 per 100,000 population in the study, however, other national studies found 368.2 in Germany (14), 1,055 in Mexico (15), 1,570 in the Netherland (16), and 4.930 in Korea (17) with an average of 427.4 years. DALYs per 100,000 population vary highly across countries which can be the result of the durations of each study, the differences in the age population pyramid between countries and the life tables chosen for each study. In this study, 99.34% of the DALYs related to YLL with the remaining due to YLD (0.66%). The study by Fan et al. conducted in several countries showed that 96.22% of DALYs are attributable to YLL and the remaining attributable to YLD (18).

Our study showed DALYs in both sexes were 2,376,552 (95% UI 2341876.0–2390969.4), equivalent to 3,061 per 100,000 in males and 1,068,471 (95% UI 1052378.6–1074780.8) equivalents to 2,573 per 100,000 in females, so results were higher in males than females; this result was also found in a recent United States study (19). DALYs increased significantly in populations above age 65. This biological inequality suggests that the higher age groups of both males and females are at a higher risk, particularly of mortality from COVID-19.

Standards-based on population size and sex-specific DALYs estimates linked to COVID-19 in several studies indicate that men bear a higher burden than women. Such a pattern has been seen in a range of disparate age groups. The age pattern of DALYs in different studies suggests the highest DALYs rate was detected in the age group 71–80 in India (20), 80–89 years in Korea (17), Ireland (21), Italy (22), and at the global level.

The findings demonstrated YLL per 100,000 population was 2842.12, this equates to 1530.22 per 100,000 years in males and 1060855.68 (95% UI 1051019.68–106988.03) is equivalent to 1248.52 per 100,000 in the females.

In general, the YLL rate is higher in males compared to females. Considering the results of previous research in 81 countries (23), for instance, the ratio of YLL females to males for COVID-19 ranges from near parity, like in Canada or Finland, to more than twice in Peru or 4-fold like in Taiwan. An example of this is the study conducted by Min-Woo et al. in Korea that showed females have lower YLL rates than males in all age groups except the age group 30–39 years (17). Likewise, the study by Yousefi et al. in Khorasan-Razavi province, Iran showed that males had greater YLLs than females except for in age-group 45–64 years. This pattern also applies to the studies reporting YLL rates per death. One study in Iran estimated the average YLL rate associated with COVID-19 deaths was 18,761 per 10,000 population in men and 16,385 per 10,000 population in women (24). The distribution of YLL rates among different age groups across a considerable amount of reviewed literature presents a similar pattern. Overall, there appears to be some evidence to indicate that YLL rates per given population increase with age in both sexes. The largest proportions of YLL in this study were in populations above age 45. A study by Pifarré i Arolas et al. indicated the opposite pattern in low- and middle-income countries, where a large proportion of the YLL is attributable to people dying at ages 55 and younger (23).

According to the results, DALYs due to YLD was 0.66%. The ratio of male YLD rates (per 1,000/100,000) to female YLD rates for COVID-19 ranges from 0.68 in Korea (17), 1.05 in Germany (14), to 1.66 in India (20). Based on the study results, both sexes had the same number of YLD (13.6 and 13.7 per 100,000 population in men and women, respectively). Some studies have revealed that the YLD rates are higher among men compared to women. YLDs per 100,000 persons represent 8 years in India (20). In our study, the highest amount in both sex groups was in the age group of 30–44 years, and then 45–59 years, and the lowest was in the age group of less than 15 years. Although some discrepancy was seen in the distribution of YLD across age groups in both sexes, the findings suggest that older age groups compared to the younger ones’ experience larger YLDs. In Korea, the YLDs rate per 100,000 population was highest in people aged 20–29 years followed by those aged ≥80 years, 50–59 years, and 60–69 years. One study in Ireland has found the proportion of YLDs was the highest in those aged 25–44 years in both sexes (17).

The WHO notes that all summary measures of population health involve explicit or implicit social value choices. The DALYs itself is a measure of the gap between the actual health status of a population and an ideal or reference status. Age weights are the most controversial value choice built into the DALYs. Some find age weights unacceptable on equity grounds (every year of life is of equal value *a priori*), others on empirical grounds (the standard age-weights do not reflect actual social values). Studies have shown that people generally prefer a healthy year of life immediately, rather than in the future. The original GBD study produced estimates using both adjusted DALYs (3% discounting and age weighting) and age weighted DALYs (no discounting, age weighting). The WHO produced further versions of adjusted DALYs, unadjusted DALYs (no discounting, no age weighting) and discounted DALYs (3% discounting, no age weighting) between 2000 and 2012 (12).

The limitations of this study included challenging access to the data, incomplete outpatient data, and the consequent need to estimate based on data from several provinces throughout the country. Another limitation was that we did not estimate the indirect effects and the direct effects on YLD caused by long-COVID.

Most of the studies in the literature review have been conducted in countries with a history of high prevalence of COVID-19. Therefore, more geographically diverse research is necessary. On the other hand, significant heterogeneity in reported measures makes cross-study comparisons difficult. Future research directions may include longitudinal studies from other phases of the COVID-19 pandemic which will likely generate different values than the ones presented here and hence, provide a more accurate picture of the burden of COVID-19 altogether.

## Conclusion

This study showed the burden of COVID-19 had a significant impact on population health in Iran. The DALYs estimates help to make international comparisons and prioritize resources to control the pandemic. The majority of population health loss was due to mortality and the most affected were the older age groups.

## Data availability statement

The original contributions presented in the study are included in the article material. Further inquiries can be directed to the corresponding author.

## Ethics statement

The studies involving humans were approved by Jiroft University of Medical Sciences. The studies were conducted in accordance with the local legislation and institutional requirements. Written informed consent for participation was not required from the participants or the participants’ legal guardians/next of kin in accordance with the national legislation and institutional requirements. Written informed consent was obtained from the individual(s), and minor(s)’ legal guardian/next of kin, for the publication of any potentially identifiable images or data included in this article.

## Author contributions

Saied-Bokaie: Writing – original draft. SD: Writing – original draft, Project administration. AB: Investigation, Validation, Writing – review & editing. AH: Data curation, Formal analysis, Software, Writing – review & editing. EB: Supervision, Validation, Writing – review & editing. DP: Formal analysis, Methodology, Writing – original draft.
